# Interaction Studies Between *Meloidogyne Javanica and Fusarium Oxysporum* f. Sp. *lycopersici (Fol) Race 3* on Different Isolines of Tomato Cv. Tasti Lee

**DOI:** 10.2478/jofnem-2022-0018

**Published:** 2022-07-27

**Authors:** Homan Regmi, Gary E. Vallad, Samuel F. Hutton, Johan Desaeger

**Affiliations:** 1 Entomology and Nematology Department, University of Florida, Gulf Coast Research and Education Center (GCREC), Wimauma, FL 33598 United States; 2 Plant Pathology Department, University of Florida, GCREC, Wimauma, FL 33598 United States; 3 Horticultural Sciences Department, University of Florida, GCREC, Wimauma, FL 33598 United States

**Keywords:** *Fusarium oxysporum* f. sp. *lycopersici race 3*, *I-3* gene, interaction, *M. javanica*, *Mi* gene, Solanum lycopersicon

## Abstract

The *Mi* gene in tomato confers resistance to *Meloidogyne javanica, M. incognita*, and *M. arenaria*, the most common tropical root-knot nematode (RKN) species found in Florida. *Fusarium* wilt (Fol) is another major problem in Florida tomatoes which may interact with RKN and cause more plant damage. To study the interactions between RKN, Fusarium, and *Mi* in tomato, two greenhouse experiments were conducted. Both experiments used different isolines (with and without *I-3* and *Mi* genes) of the tomato cultivar Tasti Lee^®^. In the first experiment, all four isolines were subjected to two levels of RKN (~10,000 eggs/pot and no eggs) and two levels of Fol (1000 cc soil with 1,000 cfu/g at planting and no Fol), both applied at planting. In the second experiment, the two isolines without *I-3* were exposed to the same two levels of RKN as described above and three levels of Fol (50 ml Fol with 1×10^6^ cfu/m at planting, at 10 DAT, and no Fol). Fol reduced root-knot infection and reproduction when both Fol and RKN were inoculated at planting but not when Fol was inoculated 10 days later. Plant damage from Fol was exacerbated in the presence of RKN, especially when both pathogens were present at planting. Isolines with *I-3* grew better in Fol-inoculated soil but had no effect when Fol and RKN were both present. Isolines with *Mi* gene reduced RKN infection and reproduction but did not affect plant damage caused by Fol. In summary, while RKN reproduction was reduced in the presence of Fol, the overall plant damage was more severe when both pathogens were present.

Root-knot nematodes (RKN; *Meloidogyne* spp.) are cosmopolitan in distribution and are the most important plant parasitic nematodes in the world (Jones et al., 2013). Three apomictic species (*M. incognita, M. arenaria*, and *M. javanica*) along with the facultative parthenogenetic species (*M. hapla*) contribute to >95% of crop losses caused by RKN (Sasser and Freckman, 1987). All four species of RKN cause damage to tomato with estimated yield losses as high as 40% in warm climatic regions (Lamberti, 1979; Williamson and Hussey, 1996; Koenning et al., 1999; NASS, 2012). *Meloidogyne* spp have been considered as one of the most serious pests in the Florida vegetable production system including tomato (Rich et al., 2003). Since the RKN infestation is more severe in sandy soils, where larger pore spaces are present in the soil which provides easy movement of the infective juveniles through the soil (Taylor and Sasser, 1978), Florida vegetable fields can be considered as a hub for this serious pest.

Host resistance in tomato against RKN is well known and is governed by a single dominant gene, *Mi*, which confers resistance to *Meloidogyne incognita*, *M. arenaria*, and *M. javanica* (Gilbert and McGuire, 1956; Robert and Thomason, 1986). Although the *Mi* gene provides good protection against several RKN species, it is ineffective against some others such as *M. hapla* and *M. enterolobii*. Also, emergence of virulent strains of southern RKN (*M. incognita*) (Kaloshian et al., 1996), the controversial issue of heat sensitivity (Dropkin, 1969; Abdul-Baki et al., 1996; Williamson, 1998; Verdejo-Lucas et al., 2013; de Carvalho, 2015) and possibility of yield drag are some major drawbacks associated with the *Mi* gene. Recent field trials in Florida showed no breakdown of *Mi* resistance during fall and spring seasons and a consistent and significant reduction in root damage and population build-up of *M. javanica* (Regmi and Desaeger, 2020).

RKN not only cause direct crop loss but also may cause indirect loss by providing entry points for other plant pathogens such as bacteria and fungi. Several researchers reported interactions between root-knot species and soil-borne pathogens like *Fusarium oxysporum* f. sp. lycopersici (Fol) in tomato and several other formae specialis in crops like cotton, pea, and chickpea (Mai and Abawi, 1987; Evans and Haydock, 1993; Back et al., 2002). Fol is one of the most devastating soil-borne fungal diseases and can cause great yield loss on susceptible tomato varieties, especially during warm seasons when both the air and soil temperatures are high (25–27°C) (Jones et al., 2014). According to their ability to infect a set of differential cultivars carrying distinct resistance loci, the virulence profile of Fol has been grouped into three races viz. race 1, 2, and 3. Race 1 was prevalent since the late 19^th^ century while race 2 was first reported in 1945 from Ohio, USA (Biju et al., 2017). Fol *race 3* (Fol *3*) was first discovered in 1979 in Australia (Grattidge and O’Brien, 1982), then in Florida in 1982 (Volin and Jones, 1982), and now is found worldwide. While most commercial tomato cultivars now have resistance against Fol races 1 and 2 (*I* and *I-2* genes), this is not the case for Fol *race* 3 (Takken and Rep, 2010). Except for production areas where Fol *3* is a problem, cultivars that have the *I-3* gene are often less popular among growers, likely because of the increased susceptibility to bacterial spot and issues with reduced fruit size (Hutton et al., 2014; Chitwood-Brown et al., 2021).

Both root-knot and *Fusarium* wilt are very common in Florida tomato fields and are considered two of the most damaging soil-borne pathogens. They can often be found together in the same field, causing tomato plants to die early. Soil fumigation is a standard practice used in the management of both these pathogens. While *Mi* tomato cultivars could be a useful tool for tomato growers in Florida, they are not commonly planted, due to a lack of appropriate cultivars and the perception that nematodes can be effectively managed with soil fumigants.

To better understand the complex interactions among RKN, Fol, and *Mi*, the following studies were conducted on four isolines of a single tomato cultivar, cv. Tasti Lee, to evaluate how the *Mi* gene performs when both RKN and Fol are present. Tasti Lee is a popular Fol *3*-resistant tomato cultivar that was developed at the University of Florida and is known for its superior taste and appearance (Scott et al., 2008; Baldwin et al., 2015; Li et al., 2018). For the purpose of our experiments, new isolines of Tasti Lee with and without the *I-3* and *Mi* genes were created.

## Materials and Methods

### General procedure

The experiments were conducted between May 2019 and April 2020 at the University of Florida’s Gulf Coast Research and Education Center (GCREC), Wimauma, Florida. Both experiments evaluated the interactions between *Meloidogyne javanica* and *Fusarium oxysporum* f. sp. lycopersici (Fol) *race* 3 using isolines of the tomato cultivar Tasti Lee. The experiments are analyzed separately as the second experiment was slightly modified to reduce the high disease pressure that was noted in the first experiment. In both greenhouse experiments, the RKN inoculum was obtained from a pure culture of *Meloidogyne javanica* that was maintained on tomato (cv. Florida 47) in the greenhouse. Eggs were extracted from infected tomato roots using 10% commercial bleach and four minutes of hand shaking (Hussey and Barker, 1973).

Tomato seedlings were grown on bleach-sterilized Styrofoam trays (128 seedlings) with steam-pasteurized potting mix and a thin layer of vermiculite on the top. Tomato seedlings (6-week-old seedlings in experiment 1 and 4-week-old seedlings in experiment 2) were planted in 1,000 cc volume plastic pots with an outside diameter of 12.5 cm. Each pot was filled with steam-pasteurized soil at 70°C for 12 h using SST-15 1/8 cubic yard 120v Soil Sterilizer (Pro-Grow Supply Corp, Brooksville, WI, USA). The soil is classified as Myakka fine sand (Sandy, Siliceous Hyperthermic Oxyaquic Alorthod) with pH of 7.6 and 0.8% organic matter. Freshly extracted eggs were inoculated (~10,000 eggs/pot) by pipetting equal volumes (1 ml) in three 2.5 cm depressions surrounding tomato seedlings immediately after transplanting. A 20-20-20 fertilizer solution (10 g/ gallon) was provided to each plant at 100 ml/pot each week throughout the experiment. Greenhouse temperature was 26 ± 2°C with a relative humidity 60% throughout the experiments with natural light condition for both experiments.

Tomato cv. Tasti Lee (*+I-3–Mi* with Fol resistance but without RKN resistance) and its three novel isolines (−*I-3, +Mi* without Fol resistance but with RKN resistance, −*I-3–Mi* without both Fol and RKN resistance and *+I-3+Mi* = with both Fol and RKN resistance) were used for the experiments. The original Tasti Lee received *I-3* gene from the parent line ‘Fla. 7907’ (Scott et al., 2008). Other three isolines were developed at Gulf Coast Research and Education Center (GCREC), Wimauma, Florida. Tasti Lee isoline without *I-3* was developed by using non-*I-3* version of ‘Fla. 7907’. The parent line ‘Fla. 8059’ received *Mi* gene from the tomato cultivar ‘Bush Early Girl’ and was used to develop Tasti Lee isoline with *Mi* gene. In all four isolines, both genes are in the heterozygous form (personal communication).

Fol *3* sand culture was used for the first experiment, and the liquid culture was used for the second experiment. The different inoculation method in the second experiment was done to reduce the disease pressure which was very high during the first experiment. For sand culture, A *race* 3 isolate of Fol, strain GEV 1400, collected in commercial field in Florida was used to produce the inoculum. The isolate was recovered from long-term storage at −80ºC by placing on a 1/2-strength potato dextrose agar (PDA) media. After 7 days at room temperature, plugs were removed from the edge of the media and placed into liquid potato dextrose broth (PDB). PDB cultures were incubated for a week at 30°C inside an orbital incubator shaker. A 200 ml suspension of Fol liquid culture with mycelium and spores was combined with a mixture of autoclaved cornmeal (500 g) and sand (2 kg) and placed in a 20 cm × 48 cm mushroom growing bag (Shroom Supply, Brooksville, Florida) with a 0.2 μ filter. Bags were sealed and incubated for two weeks at 27ºC and were cut open and allowed to dry for three more weeks. To prepare liquid culture, PDB (24 g/liter) was used to grow a pure culture of Fol race 3. Then, 1.5 ml of pure culture of Fol, stored in −20°C, was inoculated in a 1,000 ml bottle with autoclaved sterile water. The bottle was kept inside the orbital incubator shaker (Amerex Instruments, Inc, Lafayette, CA) for one week at 25°C. The final inoculum was diluted to have 1 × 10^6^ cfu/ml. The pathogen concentration was calculated using a hemacytometer (Hansen, 2000).

### Experiment 1

The experiment was conducted from the period of May to June 2019. The original Tasti Lee and it’s three isolines as described earlier were evaluated in pots with and without RKN and with and without Fol *3* (~1,000 cfu/g soil). The experimental design was a three factorial one (4 × 2 × 2). Each treatment was replicated five times and completely randomized. Then, 10.2 g of Fol-inoculated sand (9.8 × 10^4^ cfu/g) was mixed with a kilogram of steam-pasteurized soil to obtain a Fol concentration of around 1,000 cfu/g. The Fol sand culture was mixed with the steam-pasteurized soil in a cement mixer for 10 min. The premixed soil was then divided to fill each experimental pot. Seedlings were transplanted immediately after filling the pots, and RKN eggs (~10,000 eggs/pot) were inoculated at the same time as described above.

The experiment was terminated after 30 days. Plant heights were measured every week for four consecutive weeks. Root gall ratings were conducted at the end of the experiment on a scale of 0–10 (0 = no galls and 10 = 100% of roots galled) (Zeck, 1971). Nematode eggs were extracted from each root system as described above and recorded, and reproduction factor (final population density/initial population density) was calculated. Shoot and root biomass was oven dried at 70°C for 5 days, and dry weight was recorded.

### Experiment 2

This experiment was conducted from the period March to April 2020. The experiment was conducted in the same manner as previously described with the following exceptions: only Tasti Lee isolines without Fusarium oxysporum f. sp. *lycopersici* (Fol) resistance (−*I-3*, *+Mi*, and −*I-3Mi*) were used, and two inoculation timings of Fol were included, at planting and 10 days after planting. The later FOL inoculation timing was included as this has previously shown to significantly impact the interaction between the two pathogens. Each treatment was replicated five times, and the entire experiment was established as a randomized block design. Ten extra pots were included for the isoline without *Mi* to evaluate the effect of Fol on early infection of RKN. All 10 pots were inoculated with RKN, of which five received Fol at planting, while the other five did not.

In this experiment, instead of Fol soil inoculum that was used in the first experiment, liquid culture of Fol was used for inoculation to reduce the disease pressure which was very high during the first experiment. A final concentration of 5 × 10^7^ cfu in 50 ml was applied into each pot immediately after seedling transplanting or 10 days after transplanting. RKN eggs (~10,000 eggs/pot) were inoculated at planting as described above.

The 10 extra pots were sampled at 10 days after transplanting (DAT), roots were gently washed free of soil, and RKN juveniles inside roots were stained (Thies et al., 2002). Roots of J2-inoculated plants were first cleaned in running tap water and then cleaned with 10% commercial bleach by immersing individual roots in a 50 ml beaker for 4 min. Roots were then thoroughly cleaned and further immersed in clean water for 15 min more in the beaker. The water was rinsed out, and the roots were dipped into 12.5% solution of McCormick red food color (McCormick & Co., Inc., Hunt Valley, MD). The root solution was brought to a boil and allowed to cool at room temperature. The stained roots were thoroughly rinsed with water and then destained by heating with acidified glycerol (10 drops 5N HCl + 100 ml glycerol) for 20 s. This destains the root tissues, but the J2 inside the root is still stained with red color. The root was then plated in a square-shaped petri dish (10 cm × 10 cm) to observe J2s inside the root under a stereomicroscope. The main experiment was terminated 42 DAT. Plant heights were measured every 2 weeks for six consecutive weeks. Root gall ratings were conducted at the end of the experiment as described above. Nematode eggs were extracted as described above and recorded. Shoot and root was oven dried at 70°C for 5 days, and dry weight was recorded.

### Statistical analysis

Data were analyzed using SAS^®^ PROC GLIMMIX (SAS/STAT 15.1; SAS Institute Inc., Cary, NC), where tomato cultivars, RKN, and Fusarium inoculums were constants in both experiments, and block was used as a random effect in the second experiment. Student panel command was used to check the normality and homogeneity of the data before proceeding with analysis (Kozak and Piepho, 2018). Means of data were separated by using the Least squares means (LSMEANS) statement in SAS at *P* ≤ 0.05. Plant height data were analyzed using the repeated measures function. Egg count data were analyzed using negative binomial function in SAS. The ‘ilink’ option was used to get the real mean value of each dependent variable.

## Results

### Experiment 1

**Figure 1:**
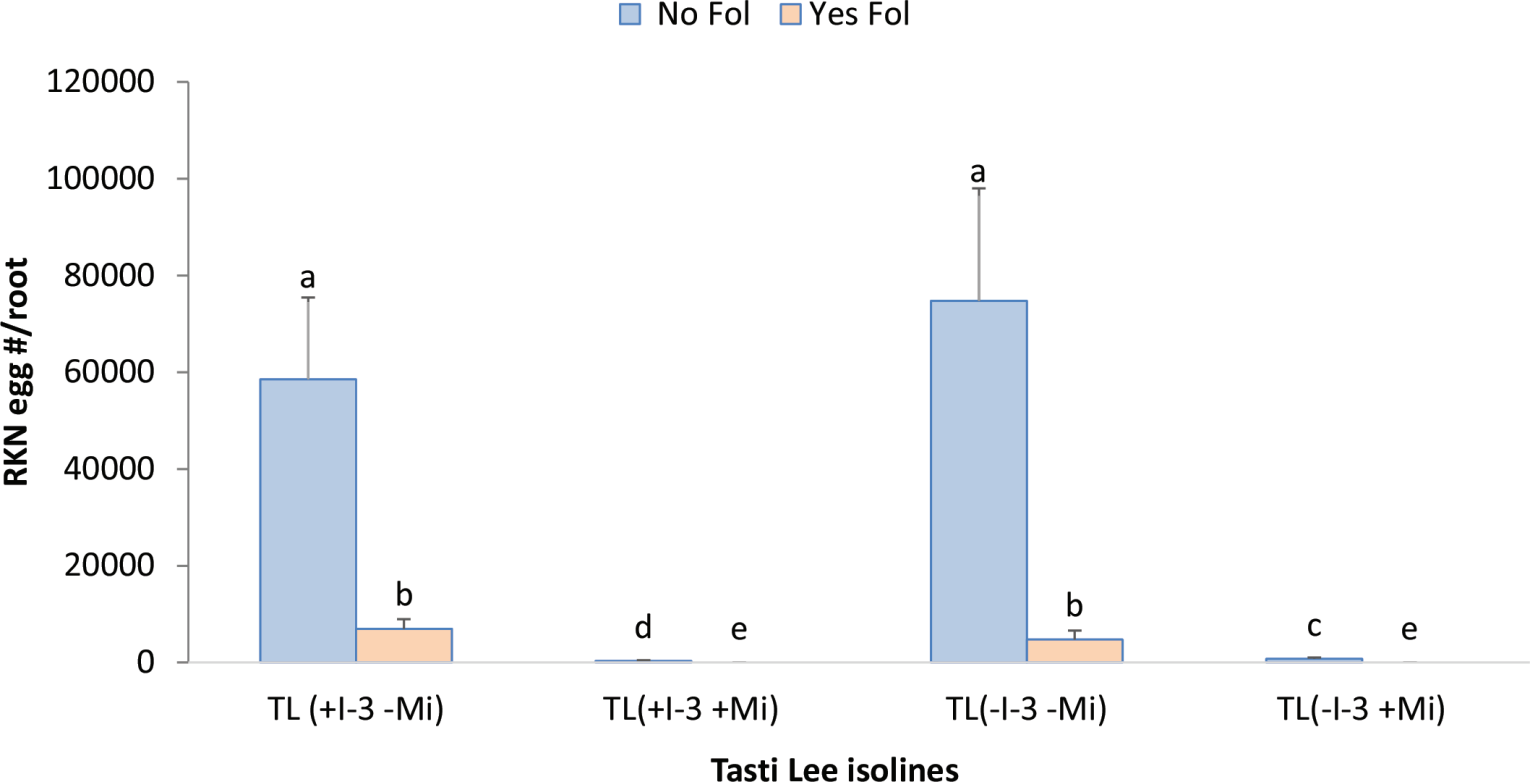
Interaction between Fol *3* and cultivar on egg production of Tasti Lee isolines (with and without *I-3* and *Mi* genes) at 28 DAT in experiment 1. TL, Tasti Lee; +, with, -, without; DAT, days after transplanting. *Note*: Although effects are grouped by genetic background, letters indicating significance apply across all means.

Fol and cultivar both showed a significant impact on RKN severity (Table 1). Root galling, egg production, and reproduction factor (RF) all were highly reduced in presence of Fol inoculum. As expected, Tasti Lee isolines with *Mi* gene significantly reduced root galling, egg production, and RF compared with the isolines without *Mi* gene. The interaction between Fol *3* and cultivars in terms of egg production was statistically significant (*P*-value = 0.003; Fig. 1) and so was RF (*P*-value = 0.004) as it depends on the final population density of the pest. RKN egg production in all Tasti Lee isolines was significantly higher when it was inoculated alone compared with when it was combined with Fol.

**Table 1:** Effect of Fol race 3 on RKN (*M. javanica*) severity in Tasti Lee isolines with and without *Mi* and *I-3* genes in experiment 1.

Factors	Level	*Gall index (30 DAT)	Eggs/root (30 DAT)	RF (30 DAT)
Fol	Yes	1.1 b	2,921 b	0.3 b
	None	2.7 a	33,580 a	3.4 a

Cultivars	TL (*+/-3, +Mi*)	0.5 b	140 d	0.01 b
	TL (−*/-3, +Mi*)	0.6 b	379 c	0.03 b
	TL (*+/-3*, −*Mi*)	3.2 a	32,736 b	3.3 a
	TL (*−/-3*, −*Mi*)	3.5 a	39,750 a	4.0 a

*P*-value	Fol	<0.0001	<0.0001	0.0004
	Cultivar	<0.0001	<0.0001	0.0008
	Fol*Cultivar	0.212	0.003	0.004

DAT, days after transplanting; RF, reproduction factor; TL, Tasti Lee; +, with; -, without. Values followed by the same letter within a column for each factor are not statistically different according to Tukey’s HSD at the 95% level of confidence (or a = 0.05). *Gall index: 0 = no visible galls in the root and 10 = whole root system galled up without any fibrous roots left and the plant is dead.

**Figure 2:**
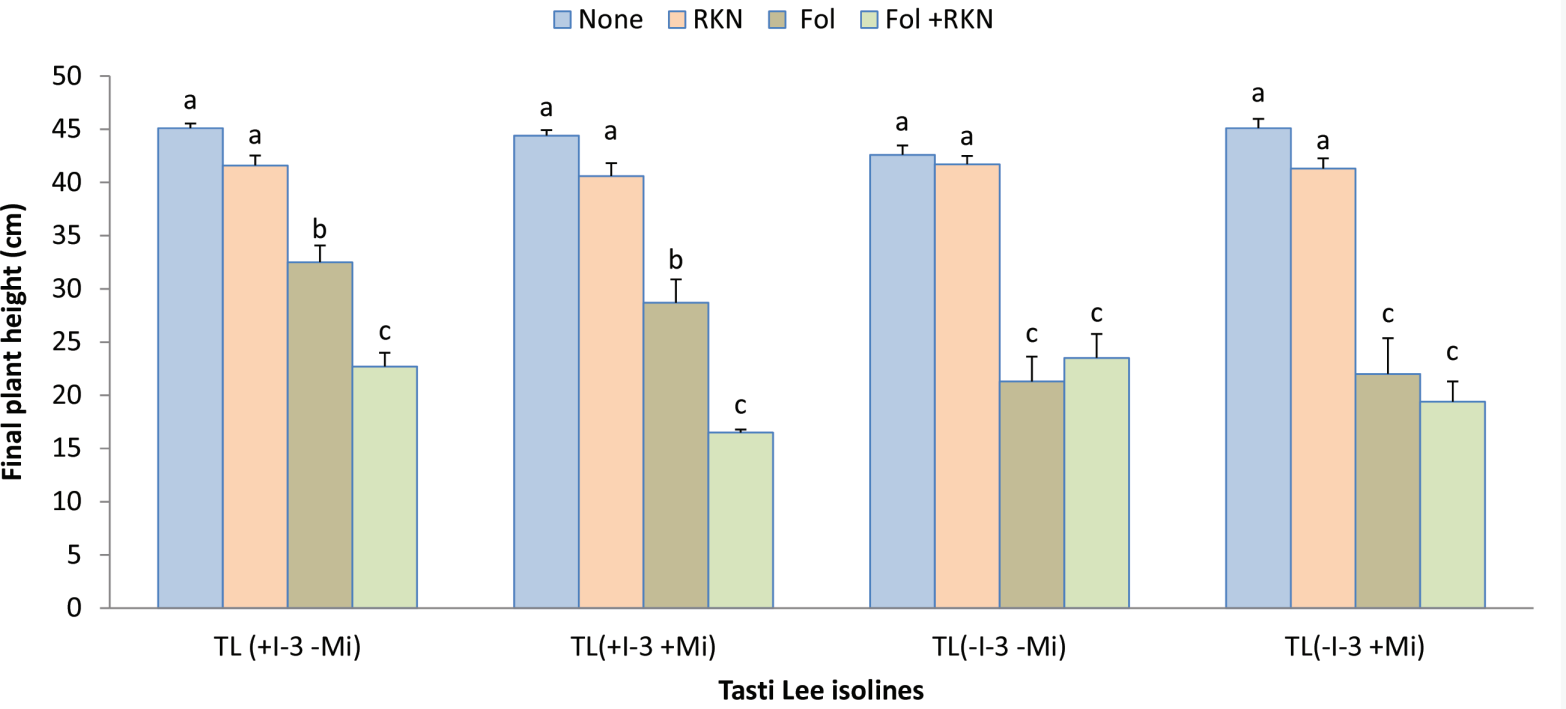
Interaction between Fol race 3, RKN (*M. javanica*), and Tasti Lee isolines on plant growth of all four Tasti Lee isolines (with and without *I-3* and *Mi* genes) at 28 DAT in experiment 1. TL, Tasti Lee; +, with, -, without; DAT, days after transplanting. *Note*: Although effects are grouped by genetic background, letters indicating significance apply across all means.

**Figure 3:**
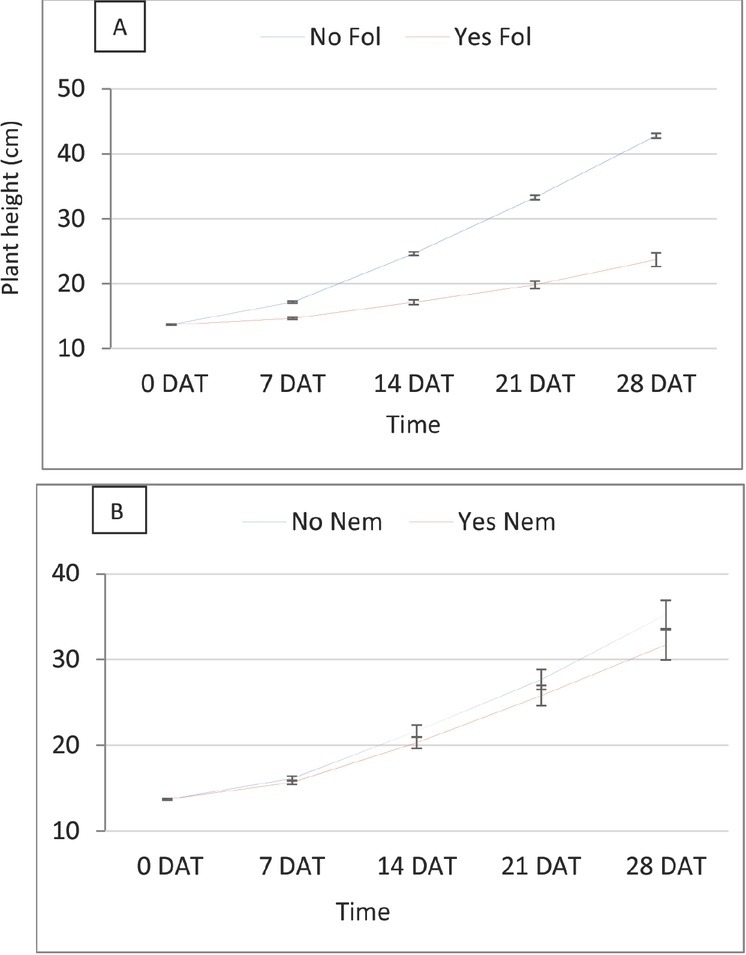
Effect of Fol (A) and RKN (B) on plant height by time in experiment 1. Fus, Fusarium wilt; Nem, root-knot nematode.

**Figure 4:**
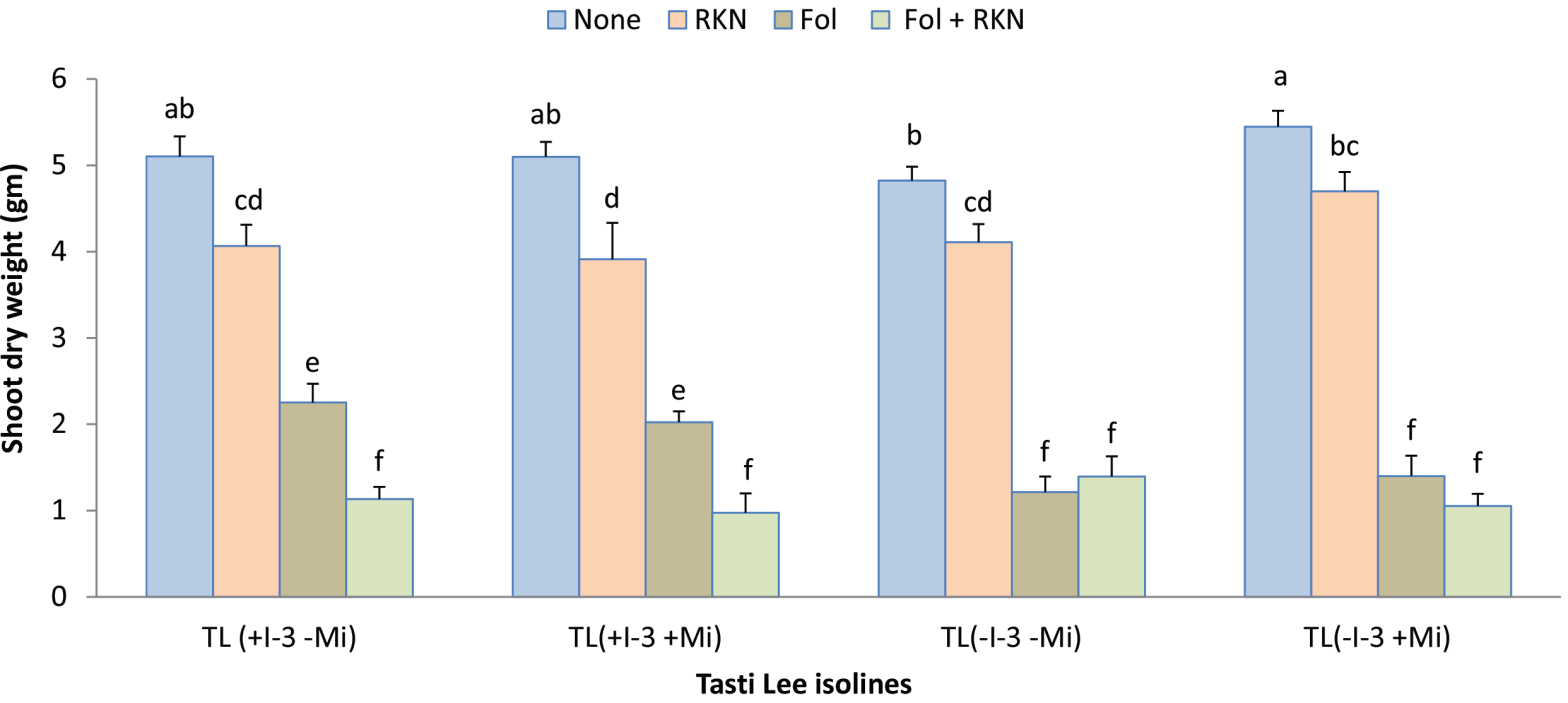
Interaction between Fol race 3, RKN (*M. javanica*), and Tasti Lee isolines on shoot biomass of all four Tasti Lee isolines (with and without *I-3* and *Mi* genes) at 30 DAT in experiment 1. TL, Tasti Lee; +, with, -, without; DAT, days after transplanting. *Note*: Although effects are grouped by genetic background, letters indicating significance apply across all means.

**Figure 5:**
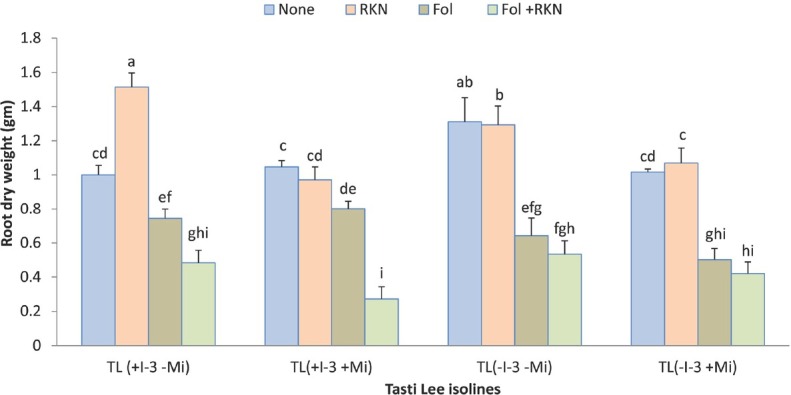
Interaction between Fol race 3, RKN (*M. javanica*), and Tasti Lee isolines on root biomass of all four Tasti Lee isolines (with and without *I-3* and *Mi* genes) at 30 DAT in experiment 1. TL, Tasti Lee; +, with, -, without; DAT, days after transplanting. *Note*: Although effects are grouped by genetic background, letters indicating significance apply across all means.

Plant height and dry biomass were negatively impacted by Fol, and RKN also slightly reduced plant height and biomass (Table 2). Interaction among RKN, Fol and cultivar for final height was statistically significant (*P*-value = 0.045; Fig. 2). RKN alone did not affect plant height much in any of the isolines, but Fol alone or in combination with RKN significantly reduced plant height in all Tasti Lee isolines relative to height given by non-treated control and RKN, with differing effect depending on presence or absence of *I-3*. Isolines with *I-3* gene had better plant height under Fol treatment alone compared to the isolines without *I-3*, but height was further reduced when Fol and RKN were inoculated concomitantly at planting; Fol alone and in combination with RKN equally reduced plant height in isolines that lacked *I-3*. Interactions between Fol and time and RKN and time for plant height were statistically significant (*P*-value <0.0001 for both interactions, Fig. 3). Both pests reduced plant height by time, and the reduction due to Fol was greater. Plant biomass, at the end of the experiment, was also significantly reduced by Fol alone and the combination of Fol and RKN (Figs. 4 and 5). Similarly, shoot dry weight was better in the isolines with *I-3* compared with the isolines without *I-3* under Fol treatment alone, but biomass was further reduced when Fol and RKN were concomitantly inoculated; likewise, Fol alone and in combination with RKN equally reduced biomass in isolines that lacked *I-3* (Fig. 4). Root dry weight was also significantly reduced by Fol and Fol+RKN treatments in all isolines (Fig. 5). Contrast analysis showed that, overall, the reduction in biomass due to Fol alone and to Fol+RKN was similar and much higher (>4 times) compared with the damage caused by RKN alone (Table 5).

**Table 2:** Effect of RKN (*M. javanica*) and Fol race 3 on plant height and biomass of Tasti Lee isolines with and without *Mi* and *I-3* genes in experiment 1.

Factor	Level	Plant height (cm)					Dry weight (gm) at 30 DAT	
		0 DAT	7 DAT	14 DAT	21 DAT	28 DAT	Root	Shoot
RKN	Yes	13.7 a	15.7 b	20.0 b	25.3 b	30.9 b	0.8 a	2.7 b
	None	13.7 a	16.2 a	21.7 a	27.6 a	35.2 a	0.9 a	3.4 a
Fol	Yes	13.7 a	14.7 b	17.0 b	19.6 b	23.3 b	0.6 b	1.4 b
	None	13.7 a	17.2 a	24.6 a	33.3 a	42.8 a	1.2 a	4.7 a
Cultivars	TL (*+I-3, +Mi*)	13.6 a	15.6 b	20.3 b	25.7 b	32.6 b	0.8 b	3.0 a
	TL (*−I-3, +Mi*)	13.9 a	15.9 ab	20.7 b	26.3 b	32.0 b	0.8 b	3.1 a
	TL (*+I-3, −Mi*)	13.8 a	16.5 a	21.9 a	27.9 a	35.5 a	0.9 a	3.1 a
	TL (*−I-3, −Mi*)	13.5 a	15.7 b	20.5 b	26.0 b	32.3 b	0.9 a	2.9 a
*P*-value	RKN	0.920	0.0246	0.0001	<0.0001	<0.0001	0.1181	<0.0001
	Fol	0.920	<0.0001	<0.0001	<0.0001	<0.0001	<0.0001	<0.0001
	RKN*Fol	0.271	0.423	0.910	0.587	0.120	<0.0001	0.134
	Cultivar	0.109	0.022	0.024	0.032	0.011	0.0005	0.277
	RKN*Cultivar	0.306	0.526	0.113	0.025	0.002	0.004	0.020
	Fol*Cultivar	0.223	0.255	0.075	0.030	0.039	0.202	0.134
	RKN*Fol*Cultivar	0.800	0.284	0.426	0.173	0.045	0.009	0.438

DAT, days after transplanting; TL, Tasti Lee; +, with; -, without. Values followed by the same letter within a column for each factor are not statistically different according to Tukey’s HSD at the 95% level of confidence (or a = 0.05).

### Experiment 2

**Figure 6:**
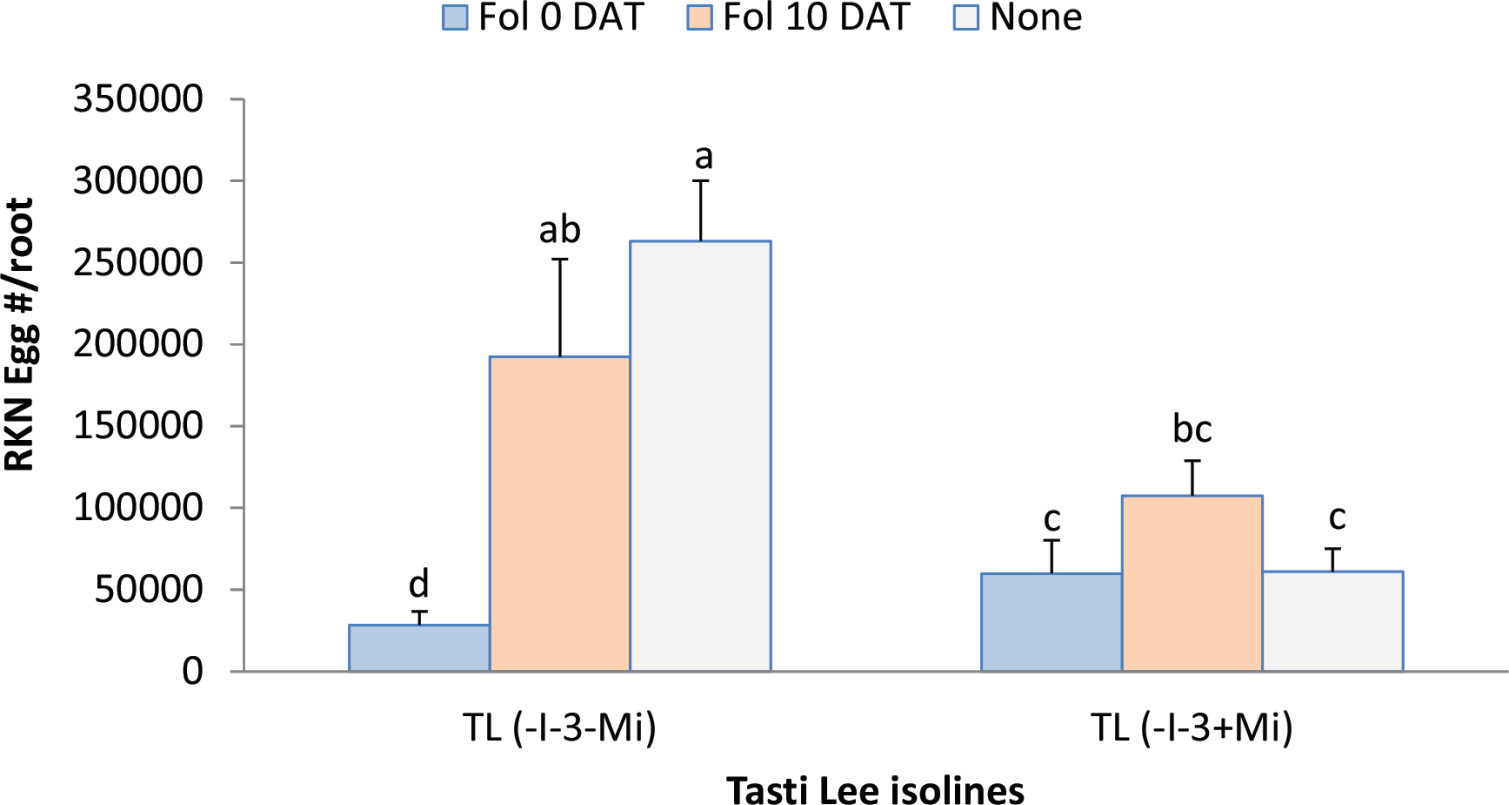
Interaction between Fol and cultivar on egg production of two Tasti Lee isolines (with and without *Mi* gene) at 42 DAT in experiment 2. TL, Tasti Lee; +, with, -, without; DAT, days after transplanting; 0 DAT, inoculated at planting; 10 DAT, inoculated 10 days after transplanting. *Note*: Although effects are grouped by genetic background, letters indicating significance apply across all means.

**Figure 7:**
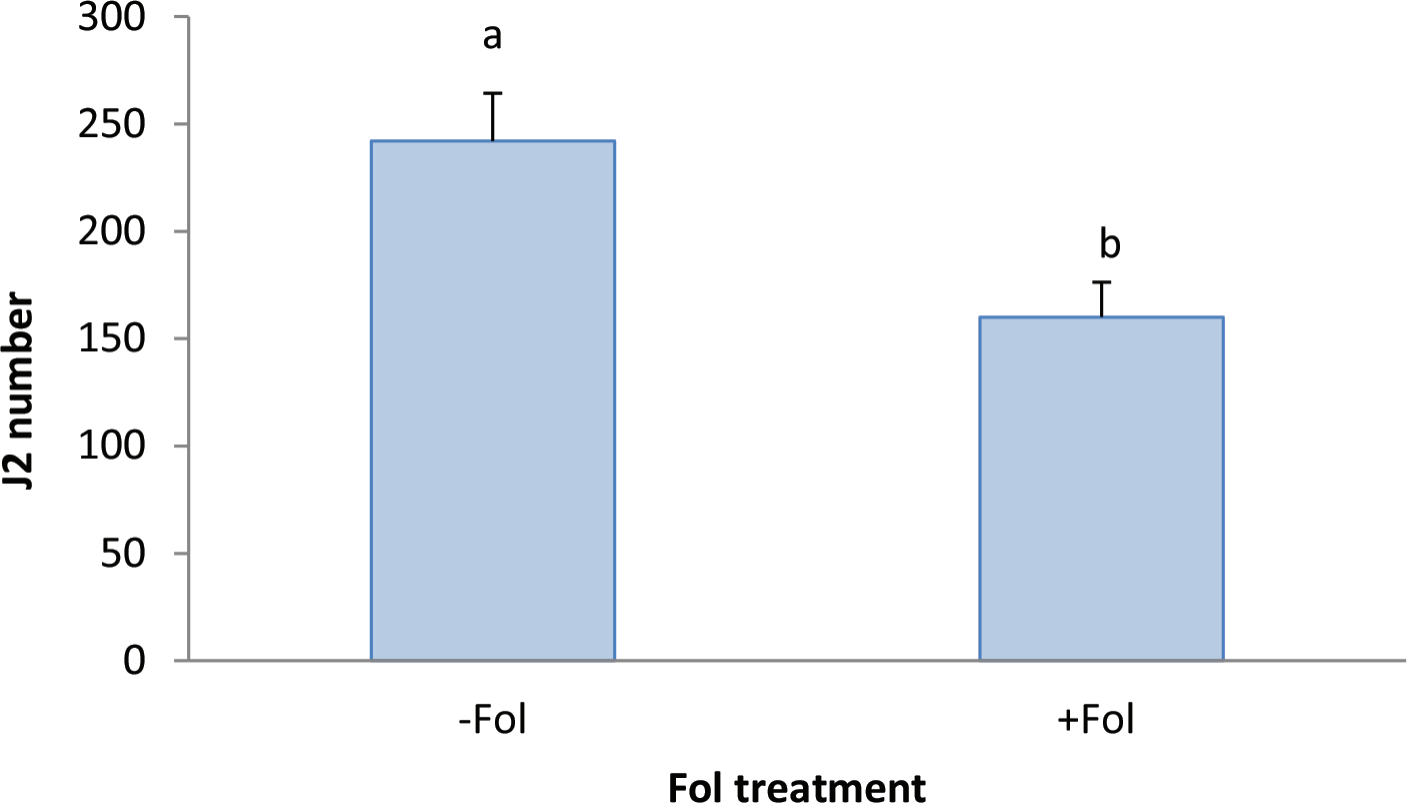
Effect of Fol on J2 penetration at 10 DAT on Tasti Lee isoline without *Mi* gene in experiment 2. J2, second stage RKN juvenile; +, with, −, without; DAT, days after transplanting; 0 DAT, inoculated at planting; 10 DAT, inoculated 10 days after transplanting.

At the end of the experiment (42 DAT), Fol inoculated at planting (0DAT) negatively impacted RKN reproduction and the isoline without *Mi* gene had higher root gall ratings, egg counts, and RF (Table 3). Gall rating tended toward being slightly reduced by Fol inoculated at 0 DAT, but differences were not statistically significant (*P*-value = 0.278). Fol inoculated at 10 DAT did not show any negative impact on RKN galling or reproduction. The interaction between Fol and cultivars for RKN egg production was statistically significant (*P*-value = 0.001; Fig. 6). In the isoline without *Mi*, egg production was significantly reduced (by 90%) when Fol and RKN were inoculated together as compared with RKN alone. But in the isoline with *Mi*, there was no significant effect of Fol on RKN egg production. Early nematode infection at 10 DAT in the isoline without *Mi* was significantly less in the plants inoculated with Fol at planting compared with plants without Fol (Fig. 7). Fol applied 10 days after transplanting did not decrease the RKN egg production in either isoline (Fig. 6). The Tasti-Lee isolines were significantly different for gall rating, egg count, and RF of *M. javanica* (Table 3). The Tasti-Lee isoline with *Mi* reduced root galls by 23% and nematode egg production and RF approximately twofold compared to the isoline without *Mi* gene.

**Table 3:** Effect of Fol race 3 on RKN (*M. javanica*) severity in Tasti Lee isolines with and without *Mi* gene in experiment 2.

Factors	Level	*Gall index (42 DAT)	Eggs/root (42 DAT)	RF (42 DAT)
Fol	0 DAT	4.5 a	44,101 b	4.4 b
	10 DAT	5.1 a	149,912 a	15.0 a
	None	5.2 a	162,088 a	16.2 a
Cultivars	TL (*−I-3*, −*Mi*)	5.6 a	161,328 a	16.1 b
	TL (*−I-3, +Mi*)	4.3 b	76,072 b	7.6 a
*P*-value	Fol	0.278	0.0002	0.002
	Cultivar	0.001	0.05	0.003
	Fol*Cultivar	0.329	0.001	0.005

DAT, days after transplanting; RF, reproduction factor; TL, Tasti Lee; +, with; -, without. Values followed by the same letter within a column for each factor are not statistically different according to Tukey’s HSD at the 95% level of confidence (or a = 0.05). *Gall index: 0 = no visible galls in the root and 10 = whole root system galled up without any fibrous roots left and the plant is dead.

**Figure 8:**
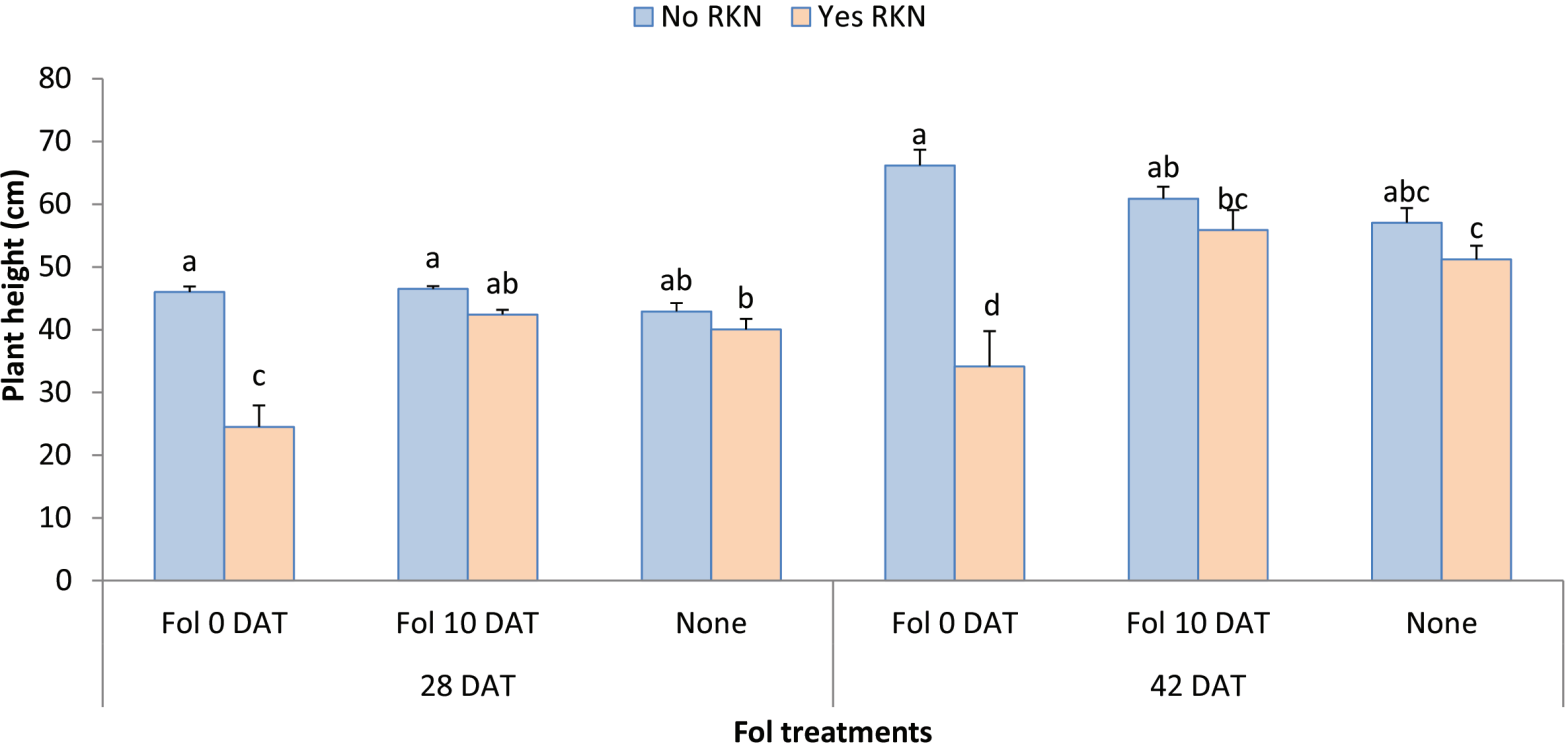
Interaction between Fol race 3 and RKN (*M. javanica*) on plant growth of two Tasti Lee isolines (without *I-3* gene and with or without *Mi* gene) at 28 and 42 DAT in experiment 2. DAT, days after transplanting; 0 DAT, inoculated at planting; 10 DAT, inoculated 10 days after transplanting.

**Figure 9:**
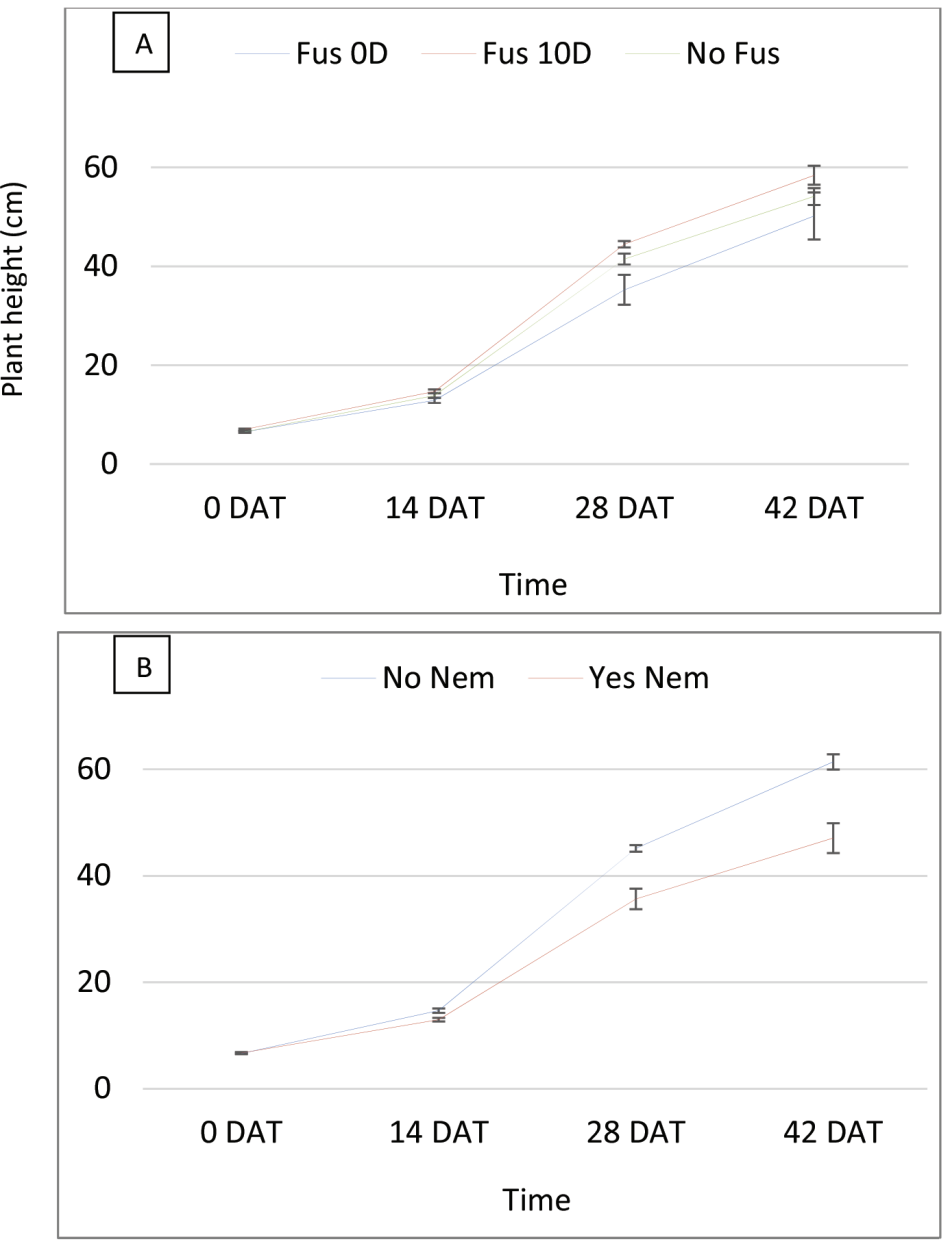
Effect of Fol (A) and RKN (B) on plant height by time in experiment 2. Fus, Fusarium wilt; Nem, root-knot nematode; 0D, 0 days after transplanting; 10D, 10; DAT, days after transplanting.

**Figure 10:**
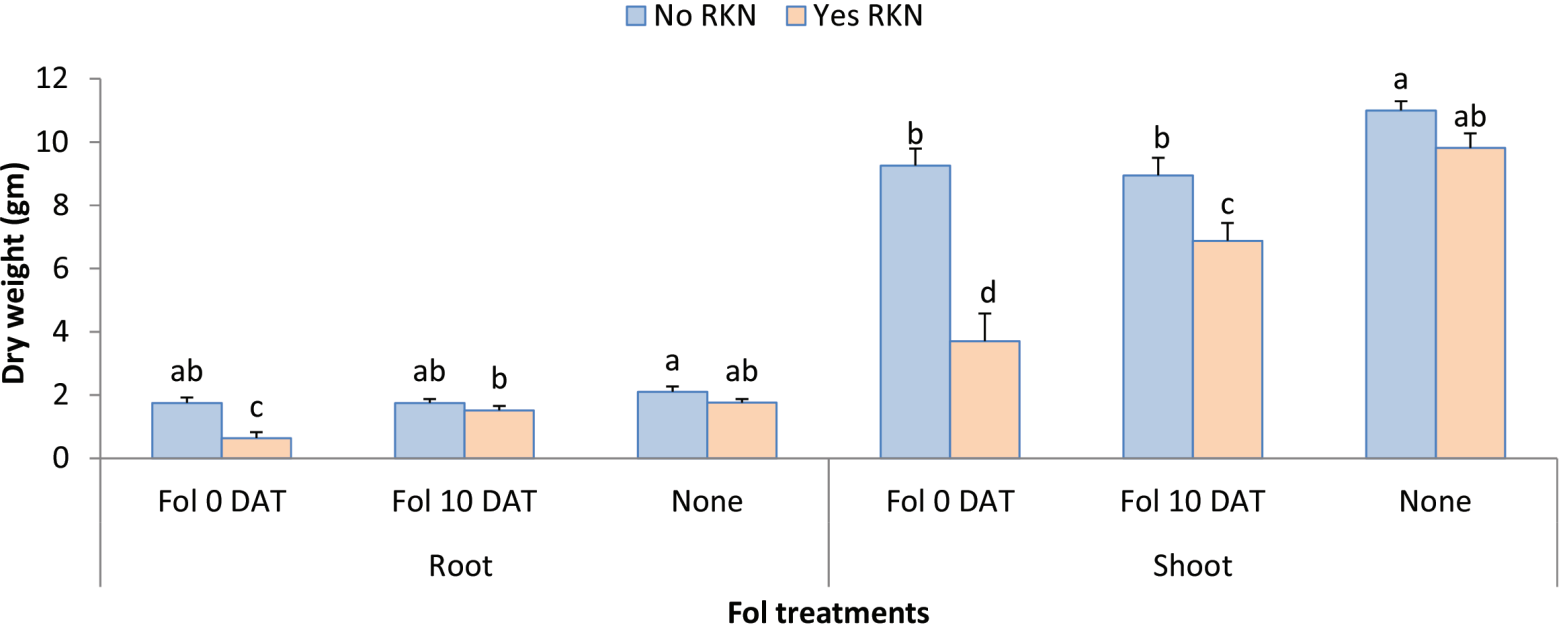
Interaction between Fol race 3 and RKN (*M. javanica*) on root and shoot biomass of two Tasti Lee isolines (without *I-3* gene and with or without *Mi* gene) at 42 DAT in experiment 2. DAT, days after transplanting; 0 DAT, inoculated at planting; 10 DAT, inoculated 10 days after transplanting.

**Figure 11:**
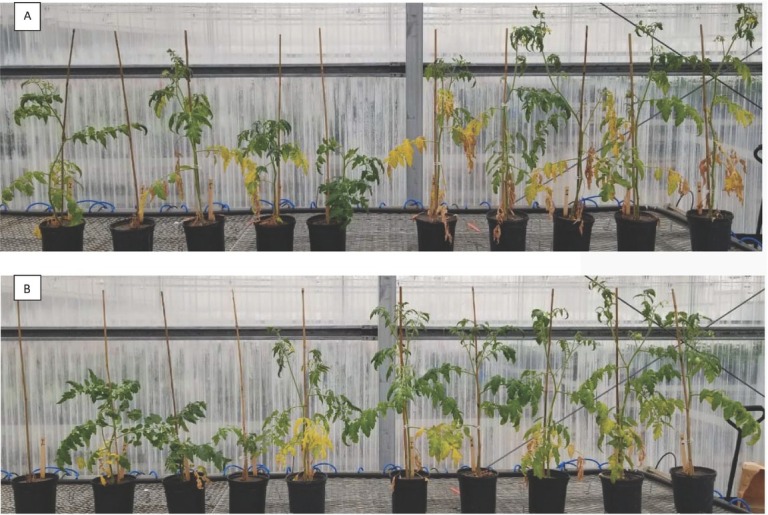
Effect of RKN and Fol and its combination on plant growth and development in tomato. (A) Tasti Lee isolines with *Mi* gene and (B) Tasti Lee isoline without *Mi* gene in experiment 2. In both pictures, five plants infected with Fol alone at right and five with Fol + *M. javanica* at left. *Photo credit*: Homan Regmi.

RKN and Fol inoculated at 0 DAT both reduced plant height and plant biomass until the end of the experiment (Table 4). Fol inoculated at 10 DAT did not negatively impact plant height but reduced shoot dry weight. There was significant interaction between RKN and Fol for plant height at 28 and 42 DAT (*P*-value: <0.0001 and 0.0001, respectively; Fig. 8). At both time points, plant height was significantly reduced by Fol inoculated at planting in presence of RKN compared with absence of RKN. *Fol* when inoculated 10 DAT did not have negative impact on plant height either with or without RKN. As in the first experiment, the interaction between Fol and time and between RKN and time for plant height was statistically significant (*P*-values 0.008 and <0.0001, respectively, Fig. 9). Both pests reduced plant height over time, but the reduction due to RKN was more prominent. Different than in the first experiment, Fol inoculated at planting slightly decreased the plant height, and the Fol inoculated at 10 DAT did not have any impact on plant height reduction. The interaction between RKN and Fol for plant biomass was also statistically significant (*P*-value = 0.0004 and 0.008, respectively; Fig. 10). Root and shoot dry weights were significantly reduced by Fol inoculated at planting in presence of RKN (Fig. 10 and Fig. 11). Fol when applied at 10 DAT also significantly reduced shoot dry weight in presence of RKN compared to its absence. Fol alone, inoculated at 0 DAT and 10 DAT, also reduced shoot biomass compared to non-treated control. The highest shoot dry weight was observed in the plants with control treatment (no RKN or Fol inoculated). Contrast analysis showed that, overall, the reduction in biomass due to Fol inoculated at 0 DAT and to RKN was similar and much lower compared with the reduction made by combination of RKN and Fol inoculated at planting (Table 5). The shoot and root biomasses were reduced by more than four and five times, respectively, by the concomitant inoculation of RKN and Fol at planting compared with the reduction caused by Fol (0 DAT) alone or by RKN alone. Reduction to shoot biomass due to the combination of RKN and Fol when inoculated at 10 DAT was also doubled when compared with Fol (10 DAT) alone.

**Table 4:** Effect of RKN (*M. javanica*) and Fol race 3 on plant height and biomass of Tasti Lee isolines with and without the *Mi* gene in experiment 2.

Factor	Level	Plant height (cm)				Dry weight (gm) at 42 DAT	
		0 DAT	14 DAT	28 DAT	42 DAT	Root	Shoot
RKN	Yes	6.8 a	13.0 b	35.7 b	47.1 b	1.3 b	6.8 b
	None	6.6 a	14.7 a	45.1 a	61.4 a	1.9 a	9.7 a
Fol	0 DAT	6.5 b	12.9 b	35.3 b	50.2 b	1.2 b	6.5 c
	10 DAT	7.0 a	14.7 a	44.5 a	58.4 a	1.6 a	7.9 b
	None	6.6 b	13.9 ab	41.5 a	54.1 ab	1.9 a	10.4 a
Cultivar	TL (−*I-3*, −*Mi*)	6.7 a	14.2 a	39.0 b	52.1 a	1.5 a	8.0 a
	TL (−*I-3, +Mi*)	6.7 a	13.5 a	41.8 a	56.4 a	1.7 a	8.5 a
*P*-value	RKN	0.479	0.0008	<0.0001	<0.0001	<0.0001	<0.0001
	Fol	0.037	0.009	<0.0001	0.049	<0.0001	<0.0001
	RKN*Fol	0.374	0.470	<0.0001	0.0001	0.008	0.0004
	Cultivar	0.919	0.151	0.049	0.105	0.219	0.278
	RKN*Cultivar	0.479	0.275	0.154	0.652	0.652	0.009
	Fol *Cultivar	0.352	0.406	0.388	0.288	0.076	0.130
	RKN*Fol*Cultivar	0.374	0.430	0.936	0.964	0.804	0.241

DAT, days after transplanting; TL, Tasti Lee; +, with; -, without. Values followed by the same letter within a column for each factor are not statistically different according to Tukey’s HSD at the 95% level of confidence (or a = 0.05).

**Table 5:** Comparisons of plant biomass in non-treated control with different combinations of RKN (*M. javanica*) and Fol race 3 in experiments 1 and 2.

Experiment 1				
Comparison	Estimated difference and level of significance			
	Shoot	*P*-value	Root	*P*-value
NTC vs RKN	0.9	<0.0001	−0.1	0.036
NTC vs Fol	3.4	<0.0001	0.4	<0.0001
NTC vs RKN+Fol	4.0	<0.0001	0.7	<0.0001

Experiment 2				

NTC vs RKN	1.2	0.120	0.3	0.106
NTC vs Fol (0 DAT)	1.7	0.024	0.4	0.1007
NTC vs RKN+Fol (0 DAT)	7.3	<0.0001	1.5	<0.0001
NTC vs Fol (10 DAT)	2.1	0.008	0.4	0.098
NTC vs RKN+Fol (10 DAT)	4.1	<0.0001	0.6	0.007

NTC, non-treated control; DAT, days after transplanting.

## Discussion

In both our experiments, the presence of Fol race 3 had a negative impact on RKN severity (root galls) and reproduction (eggs) on the tomato cv. Tasti Lee. This was most prominent when Fol and RKN were inoculated together at planting. Early root infection by root-knot J2s was reduced by half in the plants that were inoculated with Fol compared with the ones that did not get inoculated with Fol. Since RKN and Fol were inoculated together at planting, Fol may have become relatively quicker to proliferate into the root and become dominant. RKN egg hatching occurs gradually over days or weeks, and the nematode needs about 3–4 weeks to complete its life cycle (Moens et al, 2009). Significant reduction in *M. incognita* population in soil and root of carnation was observed when the plant was sequentially infected with *Fusarium oxysporum* followed by *M. incognita* where the nematode was inoculated 15 days after Fusarium inoculation and the concomitant inoculation of these two pathogens (Meena et al., 2016). The authors suggested that mycelial mat formation in the root due to Fol may have created unfavorable environmental conditions, which may have caused more RKN juveniles that were already inside roots to mold into males instead of females, reducing overall nematode reproduction (Meena et al., 2016). Lobna et al. (2016) also reported that *M. javanica* multiplication in tomato was reduced when the RKN was inoculated 10 days after *Fusarium oxysporum f. sp. radicis-lycopercisi* (FORL) was inoculated and mentioned that this might be due to physiological changes from the infection of pathogenic fungi. A similar phenomenon was reported by Hua et al. (2019) where galls produced by *M. incognita* in watermelon were reduced when the RKN species was inoculated after *Fusarium oxysporum f. sp. niveum* (FON), a soil-borne pathogen causing vascular wilt in watermelon.

In both our experiments, concomitant inoculation of Fol and RKN severely hampered overall growth and development of all Tasti Lee isolines. Fol applied at 10 DAT also reduced plant biomass, and this reduction further increased when RKN was present in the soil. However, the reduction was not as severe as the concomitant application of these two pathogens. This clearly shows that severity of Fol increases when it is combined with RKN infection, and the increase in severity is dramatic when they are present together in the soil from the beginning of tomato transplanting. Atkinson (1892) first reported the pronounced effect of RKNs in expression of *Fusarium* wilt in cotton and showed that the severity of *Fusarium* wilt increased with the infection of the nematodes. Since then, accelerated disease development in *Fusarium* wilt due to RKN infection has been reported in several crops like tomato (Jenkins and Coursen, 1957; Goode and Mcguire, 1967; Bergeson et al. 1970), pea (Davis and Jenkins; 1963), cowpea (Thomason et al., 1959), cotton (Katsantonis et al., 2003), and cucurbits (Seo and Kim, 2017; Hua et al., 2019). Recent studies on disease complex of *M. javanica* and FORL done by Lobna et al. (2016) also have shown that FORL when inoculated together with RKN or 10 days after RKN inoculation significantly reduced tomato fresh and dry weight and increased FORL severity.

Increased Fol severity and induced plant damage in presence of RKN, most of the time, are linked with the physical injuries caused by RKN juveniles. Infective RKN juveniles penetrate the root tips of tomato and enter the root system no matter whether the tomato has RKN resistance or not. This opens avenues for easy entry for the different soil-borne fungal and bacterial pathogens (Deberdt et al., 1999; Back et al., 2002). But this phenomenon may also be associated with several other physiological and anatomical changes caused by the nematode infection in the root tissue (LaMondia and Timper, 2016). Jenkins and Coursen (1957) evaluated two species of RKNs *M. incognita acrita* and *M. hapla* in combination with Fusarium wilt race 1 in tomato in greenhouse conditions. Although artificial wounding did not compromise Fol resistance in the cultivar ‘Chesapeake’, inoculation with both Fol and nematodes resulted in 100% wilting when *M. incognita acrita* was used and 60% wilting when *M. hapla* was used. It also has been reported that the plant releases more exudates during nematode infection which increases the attraction of fungus toward the root and the wounding at the same time allows the pathogens to enter the root rapidly (Back et al., 2002; Bird, 2004). As a sedentary endoparasitic nematode, RKN develops nutrient-rich feeding sites called giant cells which can a become good substrate for the fungal colonization subsequently enhancing fungal growth (c; Bird, 2004; Meena et al., 2016).

During the first experiment, although two isolines with *I-3* gene had better growth and development compared with the isolines without *I-3* gene under Fol treatment alone, the drastic reduction in plant biomass was observed in all four isolines regardless of presence or absence of *I-3/Mi* genes when plants were infected with Fol and RKN concomitantly. Davis and Jenkins (1963) also reported a similar phenomenon in pea where *Fusarium* wilt caused by *F. oxysporum* f*. pisi* was significantly influenced by *M. incognita* infection and the nematode promoted disease severity in both *Fusarium* wilt susceptible and resistant pea cultivars. Sidhu and Webster (1977) suggested that an interaction between plant and RKN could compromise the genetic resistance in a cultivar, resulting in the development of wilt symptoms. RKN may also enable nonpathogenic fungi to cause disease. In research conducted to study the disease complex caused by RKN and *Fusarium* wilt in coffee, a nonpathogenic *Fusarium oxysporum* became pathogenic when it was combined with RKN species *M. arabicida*, resulting in a complex disease symptom of corky root (Bertrand et al., 2000). But it has also been reported that Fusarium wilt resistance in tomato conferred by *I* or *I-2* gene was not affected by *M. incognita* predisposition or simultaneous inoculation (Jones et al., 1976) which contradicted the findings of Jenkins and Coursen (1957) and Sidhu and Webster (1977). In our experiment, when Fol and RKN were inoculated concomitantly, whether there was loss of resistance conferred by *I-3* gene in tomato or not was not confirmed, the synergistic effect of these two pathogens to cause damage in tomato growth was confirmed. This gives rise to new research avenues to work on the function of monogenic resistance conferred by *I-3* gene in the complex interaction among tomato, RKN, and Fol.

In both experiments, Tasti Lee isolines with *Mi* gene showed intermediate protection against *M. javanica*. This was also observed in a fall 2017 tomato field trail at GCREC where Tasti Lee isoline with *Mi* gene had more root galling and soil root-knot juvenile counts compared with other *Mi* tomatoes, Sanibel and Skyway 687 (Regmi and Desaeger, 2020). Tasti Lee isolines with *Mi* gene tend to have intermediate resistance against RKN.

Fol negatively impacted *M. javanica* root symptoms and nematode egg production especially when applied together at planting under high disease pressure. Plant fitness was severely affected when Fol and RKN were inoculated together in Fol-susceptible and resistant Tasti Lee isolines, and Fol population was increased in presence of *M. javanica*. The results suggest that Fol race 3 has an antagonistic effect on *M javanica* reproduction, but both pathogens have a synergistic effect on plant damage regardless of presence or absence of RKN and Fol race 3 resistances.
